# Initial Evidence of Mathematics Interpretation Bias Intervention Effects on College Students’ Mathematics Anxiety

**DOI:** 10.1111/nyas.70405

**Published:** 2026-09-24

**Authors:** Connie Barroso, Xiaotong Yi, Zhe Wang

**Affiliations:** ^1^ Department of Educational Psychology Texas A&M University College Station Texas USA

**Keywords:** anxiety, cognitive bias, cognitive bias modification, mathematics fluency, mathematics anxiety, mediation, undergraduate students

## Abstract

Cognitive bias modification of interpretation bias (CBM‐I) can significantly reduce negatively biased processing of anxiety‐provoking stimuli and scenarios but has not yet been tested in a mathematics context. In this study, we test the direct effects of a brief cognitive bias modification for mathematics interpretations (CBM‐MI) on threat‐related mathematics interpretation bias and both direct and indirect effects through negative interpretation mathematics bias on mathematics anxiety and performance. In an online study setting, 181 undergraduate students were randomly assigned to either the CBM‐MI condition, where they resolved ambiguous mathematics‐related scenarios in a positive way, or an active control condition where they answered neutral, nonmathematics scenarios. Results indicated strong CBM‐MI effects and significant pre‐ to post‐test changes in negative mathematics interpretation bias. CBM‐MI also had a very small direct buffering effect in increasing mathematics anxiety, and did not have any direct effects on mathematics fluency. Negative mathematics interpretation bias mediated the relation between CBM‐MI and mathematics anxiety, but not mathematics fluency. These findings provide support for moderately sized near‐transfer effects of CBM‐MI in reducing threat‐related mathematics interpretation bias and evidence of bias mediating the link between CBM‐MI and mathematics anxiety. Research should continue to investigate CBM‐MI to optimize its mathematics anxiety effects.

## Graphical abstract

We found that a brief intervention emphasizing positive interpretations of mathematics situations in college students can reduce the tendency to interpret mathematics situations as threatening. The intervention also showed small buffering effects on increases in mathematics anxiety that occurred through negative bias. No improvements in mathematics fluency were found. This study provides initial evidence that a mathematics interpretation bias intervention can reduce college students’ negative mathematics interpretation bias.

## 1 Introduction

Building a strong foundation of mathematics knowledge and skill is integral to an individual's success in their academic pursuits and future career outcomes [1, 2]. However, negative emotions can occur as students learn mathematics that can interfere with the development of mathematics skills and interest, as well as attainment of careers in mathematics‐intensive fields [3, 4]. Specifically, mathematics anxiety is a negative “feeling of tension, apprehension, or fear” that occurs when people engage with mathematics [5]. Mathematics anxiety is negatively linked with mathematics achievement [6], and thus the development and use of strategies to reduce mathematics anxiety is critical for removing emotional and cognitive barriers to performance and improving students’ STEM career outlooks.

One underlying mechanism of anxiety development is the formation of a *negative interpretation bias* in cognitive information processing, or the systematic tendency to interpret benign stimuli and situations as threatening [7]. Past research has found a strong link between negative interpretation bias and anxiety levels across many domains and contexts, including mathematics [8−11]. Cognitive bias modification for interpretation bias (CBM‐I) is an intervention approach that uses basic association principles to reduce negatively biased interpretations toward benign stimuli and also lower anxiety levels in clinically anxious samples and in the broader population [7]. Despite the promising empirical evidence and proposed theoretical accounts suggesting the utility of CBM‐I in the mathematics domain for reducing negative mathematics bias and potentially mathematics anxiety [9, 12], no intervention has yet to be developed or tested. Given these gaps in the literature, the goals of the present study are to test the generalizability of CBM‐I principles to a domain‐specific mathematics context and introduce a novel math‐specific CBM‐I paradigm as a potential new intervention technique to use in the mathematics anxiety literature.

### 1.1 Mathematics Anxiety

Mathematics anxiety is a highly prevalent and multidimensional domain‐specific emotion that exists in learners worldwide, with higher levels often reported by girls than boys [13−17]. Self‐report measures are often used to assess mathematics anxiety across dimensions that differ by context, such as mathematics evaluation contexts and classroom or learning situations [18−20]. Many research studies find moderate‐to‐strong associations between mathematics anxiety and other forms of anxiety, such as anxiety that occurs during test‐taking situations [21] as well as general anxiety [22]. Mathematics anxiety can also have broad psychological impacts, with research finding that it can predict greater levels of academic burnout [23] and reduced mental health and well‐being [10, 24]. However, it has primarily been studied in relation to other domain‐specific behaviors and psychological factors. For example, mathematics anxiety can increase behavioral avoidance of mathematics‐related tasks and interests [25−28] even when there are clear benefits and rewards tied to engagement in mathematics tasks [29], increase STEM avoidance in university students [30], and even reduce the use of effortful study strategies when studying for mathematics exams [31]. Overall, the research literature on mathematics anxiety illustrates the importance of reducing its prevalence.

Meta‐analyses indicate small‐to‐moderate negative correlations on average between mathematics anxiety and mathematics achievement, suggesting that lower levels of mathematics achievement tend to co‐occur with higher levels of mathematics anxiety [6, 32, 33]. Several prominent theories have tried to elucidate the directionality of this relation [34]. The Cognitive Interference account (aka Debilitating Anxiety) theorizes that mathematics anxiety leads to poor mathematics performance through the occupation of working memory resources that are needed for optimal learning of mathematics concepts and performance on mathematics tasks [35−37]. Conversely, the Deficit account suggests that low mathematics abilities or poor basic numerical processing skills can lead to the development of mathematics anxiety [38]. A bidirectional relation between mathematics anxiety and achievement has also been suggested, with empirical evidence indicating that each factor can influence the other over time [39]. However, not all individuals who demonstrate low mathematics ability or have a history of poor mathematics experiences develop mathematics anxiety. Importantly, theoretical and empirical research from the clinical anxiety field has identified the influence of *cognitive biases* in the development of anxiety disorders. Described in the next section, this research base can be used to further guide understanding in the mathematics anxiety field and explain why students, with or without low mathematics ability or poor mathematics experiences, develop mathematics anxiety.

### 1.2 Cognitive Biases in Anxiety Dysfunction

In the clinical anxiety literature, anxiety is suggested to be triggered by a vigilance to threat‐related schemas, which in turn guides an individual's cognitive processing and behavioral response [40]. While theorized to be an adaptive mechanism, cognitive biases can manifest themselves in this process as overreactions in perceiving ambiguous cues in the environment as potential threats and subsequently turn an adaptive survival mechanism into a maladaptive one that can lead to anxiety disorders. Interpretation bias is a specific type of cognitive bias that is characterized as the systematic tendency to interpret stimuli in a specific manner, as either positive, neutral, or threatening, with the latter interpretation being defined as a *negative* interpretation bias [41]. Considerable research suggests a close association between negative interpretation bias and anxiety in general [42, 43], as well as between domain‐specific negative interpretation bias and their corresponding anxieties (e.g., social situations, health contexts) [43−46].

In the mathematics education literature, the theoretical account by Ramirez et al. provides further domain‐specific support for the presence of mathematics‐specific interpretation bias in cognitive processing that can lead to mathematics anxiety [12]. This account hypothesizes that individuals create their own meaning of their past mathematics experiences, which becomes an internal narrative or lens through which they interpret their present mathematics experiences. For example, a student who is failing a mathematics test grade could internalize this experience to have a positive meaning or a negative one. The former internal narrative would frame present or future mathematics experiences in a positive light, whereas the latter one would lead to maladaptive interpretations of present or future mathematics experiences and lead to the development and/or activation of mathematics anxiety. This relationship may additionally be bidirectional, with mathematics anxiety subsequently reinforcing the negative interpretation and both together forming a negative perpetual cycle. Recent empirical evidence provides support for the connection between negative interpretation bias and anxiety specifically in the mathematics domain in a sample of college students [9]. Altogether, the cognitive bias account of anxiety dysfunction, interpretation account of mathematics anxiety, and empirical research findings suggest that reshaping negative interpretations of mathematics situations may lead to a reduction of mathematics anxiety.

### 1.3 Cognitive Bias Modifications for Interpretation Bias in a Mathematics Context

Cognitive bias modification for interpretation bias (CBM‐I) is a common paradigm from the clinical psychology literature that uses basic association principles to link the biased information processing of stimuli or scenarios with a certain resolution [40]. To reduce negatively biased interpretations, CBM‐I paradigms train individuals to resolve ambiguous scenarios in either a positive or a benign manner [47, 48]. Many research studies show small‐to‐moderate near transfer effects of both benign and positive CBM‐I training on assessments evaluating similar biases in cognitive processing, specifically finding reductions in biased threat interpretations and increases in positive interpretations in adult and youth samples [47, 49−53]. Thus, CBM‐I may be a promising paradigm to use in mathematics contexts to reduce the prevalence of negative mathematics interpretation biases that learners may develop during their mathematics encounters.

Additionally, some empirical evidence also suggests that CBM‐I has direct far transfer effects, so to say effects on other processes related to or beyond the directly targeted biased interpretation processing, on clinical symptoms such as general and social anxiety, depression, and even on physiological indicators of stress [54] (however, see [55] for no transfer effects). Research findings of far transfer effects suggest that CBM‐I can reduce the strength of clinical symptoms immediately after training [48, 53] and after presentation of stressors [52]. Beyond these direct effects, research has also found that the effects of CBM‐I on anxiety are mediated by reductions in negatively biased interpretation processing [56]. Overall, the empirical work applying CBM‐I paradigms to reduce biased cognitive processing and its relative success in reductions of anxiety and other outcomes further support the use of CBM‐I in mathematics contexts to reduce negative bias as well as potentially reduce mathematics anxiety and other related outcomes in a direct or indirect manner. However, no mathematics‐specific CBM‐I has been developed to date to test these possibilities.

### 1.4 The Present Study

Maladaptive cognitive biases are common and malleable, and CBM‐I principles developed in clinical contexts have the potential to generalize to domain‐specific academic settings to reduce negative academic‐related cognitive biases. The link between negative mathematics interpretation bias and mathematics anxiety provides further support that math‐specific CBM‐I may have significant implications for anxiety through the possible reduction of negative mathematics interpretation bias [9]. In the present study, we developed a novel mathematics‐specific CBM‐I paradigm to test whether CBM‐I principles can be successfully applied to mathematics contexts and produce direct effects on mathematics‐specific negative interpretation bias and either direct or indirect effects on mathematics anxiety and achievement.

To this end, we implemented a two‐group pre‐post experimental design in which undergraduate students were randomly assigned to either a mathematics‐specific CBM‐I condition, in which they completed a positive mathematics interpretation bias modification intervention, or a Control condition where they completed a non‐mathematics‐specific task (i.e., active nonmathematics control condition). We address the following set of research questions and respective hypotheses:

1. Is there evidence of near transfer effects of mathematics‐specific CBM‐I?
  1a. We hypothesize that the CBM‐I condition would have lower post‐test negative mathematics interpretation bias relative to the control condition.
  1b. We hypothesize that the CBM‐I condition would show greater pre‐to‐post reductions in negative mathematics interpretation bias relative to the control condition.
2. Is there evidence of far transfer effects of mathematics‐specific CBM‐I?
  2a. We hypothesize that the CBM‐I condition would have lower mathematics anxiety and higher mathematics achievement at post‐test relative to the control condition.
  2b. We hypothesize that the CBM‐I condition would show greater reduction in mathematics anxiety and improvement in mathematics achievement from pre‐ to post‐test relative to the control group.
3. Does the mathematics‐specific CBM‐I generate a near‐transfer effect, which in turn contributes to the far‐transfer effect?
  3a. We hypothesize that post‐test reductions in mathematics anxiety and improvements in mathematics achievement for students in the CBM‐MI condition would be mediated by decreases in post‐test negative interpretation bias between conditions.

## 2 Materials and Methods

### 2.1 Sample

A total of 183 undergraduate students were recruited to participate in our online experimental study through a university‐wide bulk e‐mail listserv at a large higher education university in the South Central region of the United States. Eligible participants were required to be at least 18 years old, actively enrolled as an undergraduate student at an academic institution, speak English, and have access to an internet‐connected computer. The study was conducted via Zoom during a scheduled 1‐h online appointment between the participant and a trained research assistant. Data were collected from the start of fall 2021 through the end of fall 2022. Two participants experienced technical glitches throughout data collection and were not included in our analyses.

Our final analytic sample was 181 participants (mean age = 20.06 years old, *SD* = 1.78; *n* = 139 female, *n* = 40 male, *n* = 2 missing gender data). Participants ranged in their school year status, with 28% freshmen, 18% sophomores, 19% juniors, and 33% seniors (2% indicated other). Regarding race, 1% of participants identified as Native Hawaiian or Other Pacific, 2% identified as American Indian or Alaska Native, 5% identified as Black or African‐American, 5% identified as Multiracial, 29% identified as Asian, and 56% identified as White (3% preferred not to indicate). Twenty‐seven percent of the sample indicated they were of Hispanic ethnicity. Participants represented students from a variety of majors across the university (list of majors presented in Table S1).

### 2.2 Procedure

At the start of the scheduled appointment time, research assistants shared a Qualtrics link for participants to use during the study via the Zoom chat feature. Informed consent was obtained on the first page of the Qualtrics survey. Participants were then asked to share their screen that contained the browser window with the Qualtrics survey so the research assistant could monitor the participant's pacing and adequate engagement in the study.

First, participants completed pre‐test surveys assessing mathematics interpretation bias, mathematics anxiety, test anxiety, and general anxiety (along with other surveys not analyzed in the present study). They also completed Form A of the mathematics fluency test. The presentation of the surveys and mathematics test was counterbalanced (i.e., surveys presented first, assessment presented second, vice versa). Participants were then randomly assigned to complete tasks in either an Experimental condition or Control condition (described in the Conditions section, 2.3.3). Afterward, participants completed post‐test measures assessing mathematics anxiety, mathematics interpretation bias, and Form B of the mathematics fluency test, also counterbalanced, and educational background and demographics questions. The interpretation bias and assigned Condition tasks were completed in PsyToolkit, an online experiment software [57]. The other surveys and tasks were completed in Qualtrics. Each student received a $10 e‐gift card as a thank you for their time and participation. This study received ethical approval from the Texas A&M University Institutional Review Board (approval #IRB2021‐0244 M).

### 2.3 Measures

#### 2.3.1 Main Study Variables

##### 2.3.1.1 Mathematics Interpretation Bias

Students completed the Word‐Sentence Association Paradigm—Mathematics Interpretations (WSAP‐MI) [9]. This paradigm was adapted from the WSAP, a commonly used measure of general interpretation bias [58]. Details about the WSAP‐MI item and measure development process can be found in Barroso et al. [9].

In the WSAP‐MI, participants complete a series of 48 trials where they are asked to quickly indicate how related they think a word and an ambiguous mathematics‐related scenario are to each other. Each trial of the WSAP‐MI has four steps. Participants are first shown a fixation point in the middle of a black screen on a computer. A sentence then appears with an ambiguous mathematics‐related scenario that a college student might experience. Next, a word appears below the sentence that disambiguates the scenario in a positive manner or a negative/threatening manner. Participants then indicate to what extent they think the word and sentence presented on the screen are related to each other on a Likert scale from 1 (*not related*) to 6 (*very related*).

There are 24 unique scenarios that describe college students’ mathematics experiences related to mathematics class, learning, solving problems, test‐taking, homework, peers, and instructors (e.g., *Your teacher says you will be taught something new in mathematics class today*. *You walk into your mathematics class and you notice how you feel.; You are halfway done with your mathematics homework*.). Each scenario is randomly repeated two times across the trials, with 24 trials paired with a positive word (e.g., *attentive; calm; accurate*) and the other 24 trials paired with a negative word (e.g., *intimidating; dread; wrong*). Trials are presented in the same order for all participants. The resulting 1–6 rating from each trial provides a measure of the participant's tendency to resolve ambiguous mathematics situations in a negative or positive manner. Specifically, positive word pair ratings indicate the extent to which participants agreed that positive resolutions of ambiguous mathematics scenarios were related, whereas negative word pair ratings indicate the extent to which participants agreed that threatening or negative resolutions of ambiguous mathematics scenarios were related.

To obtain our measure of negative mathematics interpretation bias, we first averaged ratings from the positive pair trials and negative pair trials separately to create average positive scores (pre‐test Cronbach's α = 0.89, post‐test Cronbach's α = 0.92) and average negative scores (pre‐test Cronbach's α = 0.93, post‐test Cronbach's α = 0.95), respectively. A negative mathematics interpretation bias score was calculated following the process done in past research by subtracting the positive average score from the negative average score for each participant (pre‐test Cronbach's α = 0.93, post‐test Cronbach's α = 0.95) [59, 60]. This method is used to calculate a pure negative bias score, as it takes into account simultaneous levels of positive bias a person holds alongside their negative interpretations. Higher scores above 0 indicate a higher negative bias, and negative scores below 0 indicate that there is a lower negative bias.

##### 2.3.1.2 Mathematics Anxiety

Participants completed the 9‐item Abbreviated Mathematics Anxiety Scale (AMAS) [61]. In this scale, participants are asked to rate how much anxiety they would feel in the situation described in the statement that is presented to them, on a scale from 1 (*low anxiety*) to 5 (*high anxiety*). Four of these items assess anxiety related to mathematics learning situations (i.e., *Listening to a lecture in mathematics class*; *Watching a teacher work an algebraic equation on the blackboard*). Five items assess anxiety related to test‐taking that occurs in mathematics contexts (i.e., *Being given a “pop” quiz in mathematics class; Thinking about an upcoming mathematics test 1 day before*). A mathematics anxiety score was obtained by calculating the mean rating across all nine items (pre‐test Cronbach's α = 0.88, post‐test Cronbach's α = 0.89).

##### 2.3.1.3 Mathematics Fluency

Students completed the timed, 1‐min multiplication quiz from Conlon et al. that included 40 single‐digit multiplication problems (i.e., 8 × 7) [62]. Items were presented on the screen in rows of five, with eight rows altogether, and no item included 0 or 1 as an operand. An automatic timer was included at the bottom of the page below the eight rows of items. Two forms (Forms A and B) were used for this quiz. The forms were identical to each other with regard to the multiplication problems presented. However, the order of items presented in each row of Form B was reversed to that of Form A, with the last item of the row presented first, the second‐to‐last item presented second, and so on.

Two scores were initially calculated from the multiplication quiz. One score was calculated by adding up the number of correct answers solved in the minute‐long test, reflecting mathematics fluency/efficiency [62]. The other score was calculated by dividing the number of answers an individual participant answered correctly by the number of items they were able to complete, reflecting mathematics accuracy percentage. However, the average accuracy percentages at both the pre‐ and post‐test were high (pre‐test: *M* = 95.85%, *SD* = 5.36; post‐test: *M* = 96.24%, *SD* = 4.82) and highly skewed (pre‐test skewness: −2.33; kurtosis: 9.27; post‐test skewness: −2.42; kurtosis: 8.92). Scores ranged from 61.90% to 100%, with between 40% and 43% of participants receiving an accuracy score of 100% (pre‐test *N* = 78; post‐test *N* = 72). This indicated a ceiling effect with very limited variability for the accuracy percentage variable of mathematics achievement. Due to the restricted variance, the accuracy percentage variable was excluded from further analyses (descriptive statistics are reported here for transparency). We focus our mathematics achievement analyses on the mathematics fluency variable.

#### 2.3.2 Covariates

##### 2.3.2.1 Gender

Participants were asked to respond to the question *What is your gender*? by selecting one of the following options: *Female* (coded as 0), *Male* (coded as 1), *Not listed (specify)*, or *Prefer not to say*.

##### 2.3.2.2 Age

Participants were asked to respond to the question *What is your age*? and selected their answer from a drop‐down menu that listed age options from 18 to 75 years old.

#### 2.3.3 Conditions

**FIGURE 1:**
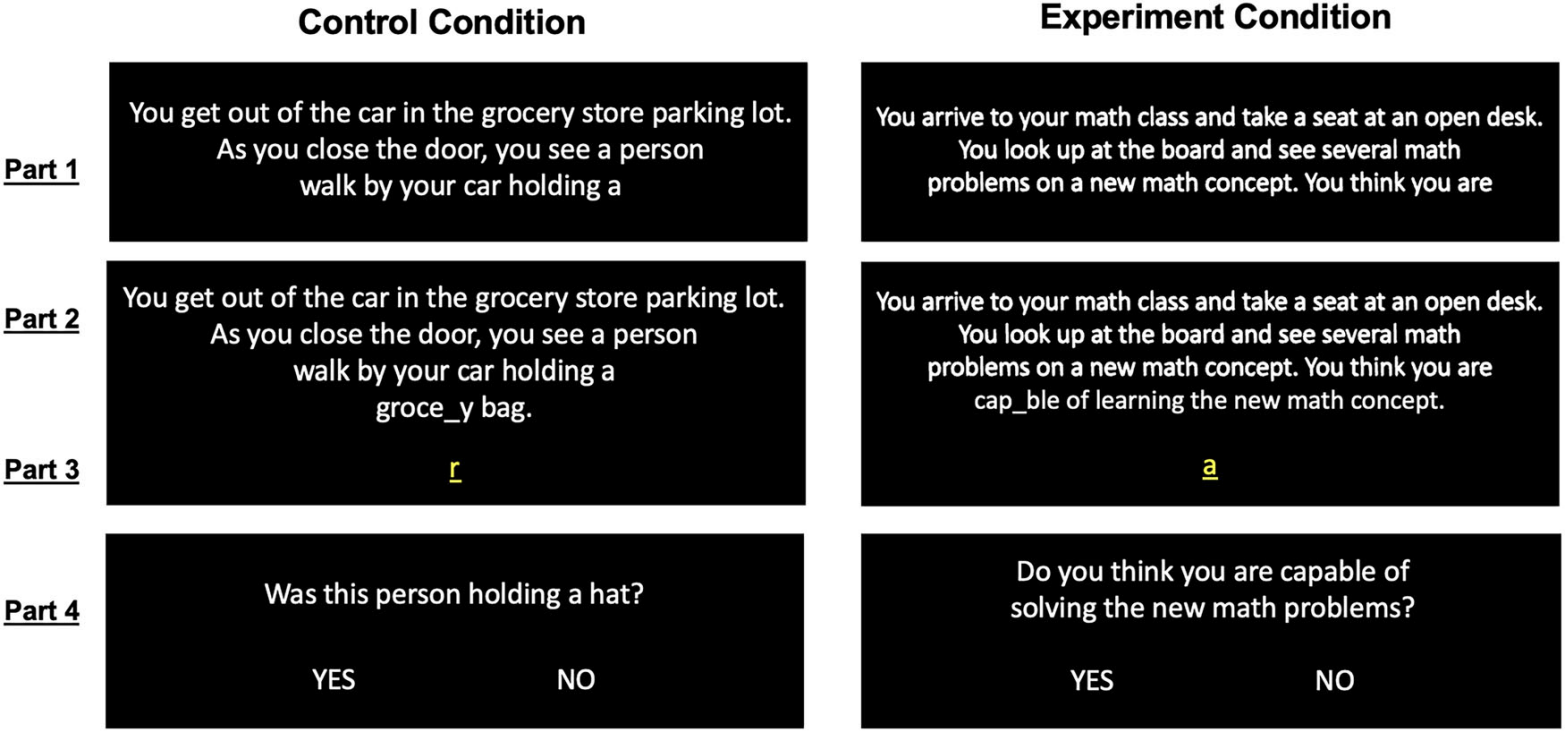
Example of the four parts of a trial item in the scenario task for the control condition and the cognitive bias modification‐mathematics interpretation condition.

Participants in both conditions used the same four‐part protocol for their respective scenario tasks (Figure 1). First, an incomplete ambiguous scenario is presented on the computer screen. Participants click the space bar to complete the sentence, which resolves the ambiguous scenario in a specific direction. After 5 s, participants are asked to type in a letter that one of the words in the scenario is missing. They are then presented with a simple comprehension question designed to solidify the resolution of the previously ambiguous scenario, to which they answer either “Yes” or “No” by typing the letter “e” or “o” on their keyboard, respectively. Participants receive accuracy feedback based on their response (*You made a mistake* for incorrect answers or *Well done* for correct answers) and continue to the next item in the scenario task once they have responded correctly.

##### 2.3.3.1 Experimental Condition

Participants in the experimental condition were asked to complete a researcher‐developed scenario task called the Cognitive Bias Modification—Mathematics Interpretations (CBM‐MI), adapted from those outlined in Mathews and Makintosh, and Hawkins and Cougle [7, 63]. Participants engaged with a series of six sequential mathematics‐related scenarios that described learning a new mathematics concept in class, working on mathematics problems at home, working on a mathematics assignment in class, studying for a mathematics test, taking a mathematics test, and finding out a grade on a mathematics test. The whole task consisted of 40 individual trial items.

##### 2.3.3.2 Control Condition

Participants assigned to the control condition engaged with a series of three scenarios describing typical, nonmathematics experiences that an average adult might encounter: grocery shopping, going to the park, and eating dinner at a restaurant. Ambiguous scenarios were neutrally resolved. Each series of scenarios included either 10 or 15 trial items to complete, for a total of 40 items to complete in the task.

### 2.4 Transparency and Openness

An earlier version of the data in this manuscript was presented in a symposium at the 2024 American Educational Research Association Meeting. This study was not preregistered. Materials and code for reproducing all analyses are open and available online via the Open Science Framework (https://osf.io/8t7zh/).

## 3 Results

Analyses were conducted in SPSS (version 29.0). Average scores for pre‐test and post‐test study variables were calculated based on available data (see Supplementary Materials for item‐level missing data). All variables except for gender (*n* = 2) and the post‐test negative mathematics interpretation bias score (*n* = 2 missing due to technical glitches in post‐test experimental software) had average scores calculated. Missing data in analyses with gender and post‐test negative mathematics interpretation bias variables were handled with listwise deletion.

Descriptive statistics for study variables are presented in Table 1. All variables except age had skewness and kurtosis values under the absolute value of 2 [64]. Significant differences by Condition for pre‐test study variables and covariates were found only for the participant mean age (*F*(1,179) = 8.58, *p* = 0.004), with the Control group including a sample of older participants on average (Table S2). Correlation coefficients between the pre‐test study variables are included in Table S3.

**TABLE 1:** Descriptive statistics for study variables.

Variables	Sample size	Actual Min−Max	Mean	SD	Skewness	Kurtosis
*Pre‐test variables*						
Negative mathematics interpretation bias	181	−4.29 to 3.13	−0.43	1.30	0.12	0.25
Mathematics anxiety	181	1.00–4.78	2.53	0.75	0.49	−0.14
Mathematics fluency	181	6–40	25.91	7.53	0.10	−0.54
Age (years)	181	18–31	20.06	1.78	1.53	6.69
Gender^a^	179	0 or 1	—	—	—	—
						
*Post‐test variables*						
Negative mathematics interpretation bias	179	−4.58 to 3.17	−0.77	1.49	−0.13	0.13
Mathematics anxiety	181	1.00–4.44	2.58	0.76	0.26	−0.62
Mathematics fluency	181	11–40	29.03	6.60	−0.10	−0.43

Abbreviations: Min, minimum; Max, maximum; SD, standard deviation; Skew, skewnesss, Kurt, kurtosis.

^a^Female = 0, male = 1.

In addition to analyses with the negative mathematics bias score, we also present results for positive and negative mathematics interpretation trial average scores in Tables S3−S9. These analyses show the same pattern of results that will be described as the negative interpretation bias score (i.e., increases in positive interpretations, decreases in negative interpretations). Bias score results are reported in the main text to simplify the presented results and align with past work using this scale [9].

Finally, effect sizes are interpreted according to Cohen [65]. Standardized coefficients from linear regression analyses are interpreted as small if *β* is equal to 0.10 through 0.29, medium if it is equal to 0.30 through 0.49, and large if it is greater than 0.50. Partial *η* ^2^ from repeated measures ANCOVA results are interpreted as small if partial *η* ^2^ is equal or greater than 0.0099, medium if it is equal or greater than 0.0588, and large if it is equal or greater than 0.1379.

### 3.1 Main Analyses for Research Questions 1 and 2

**FIGURE 2:**
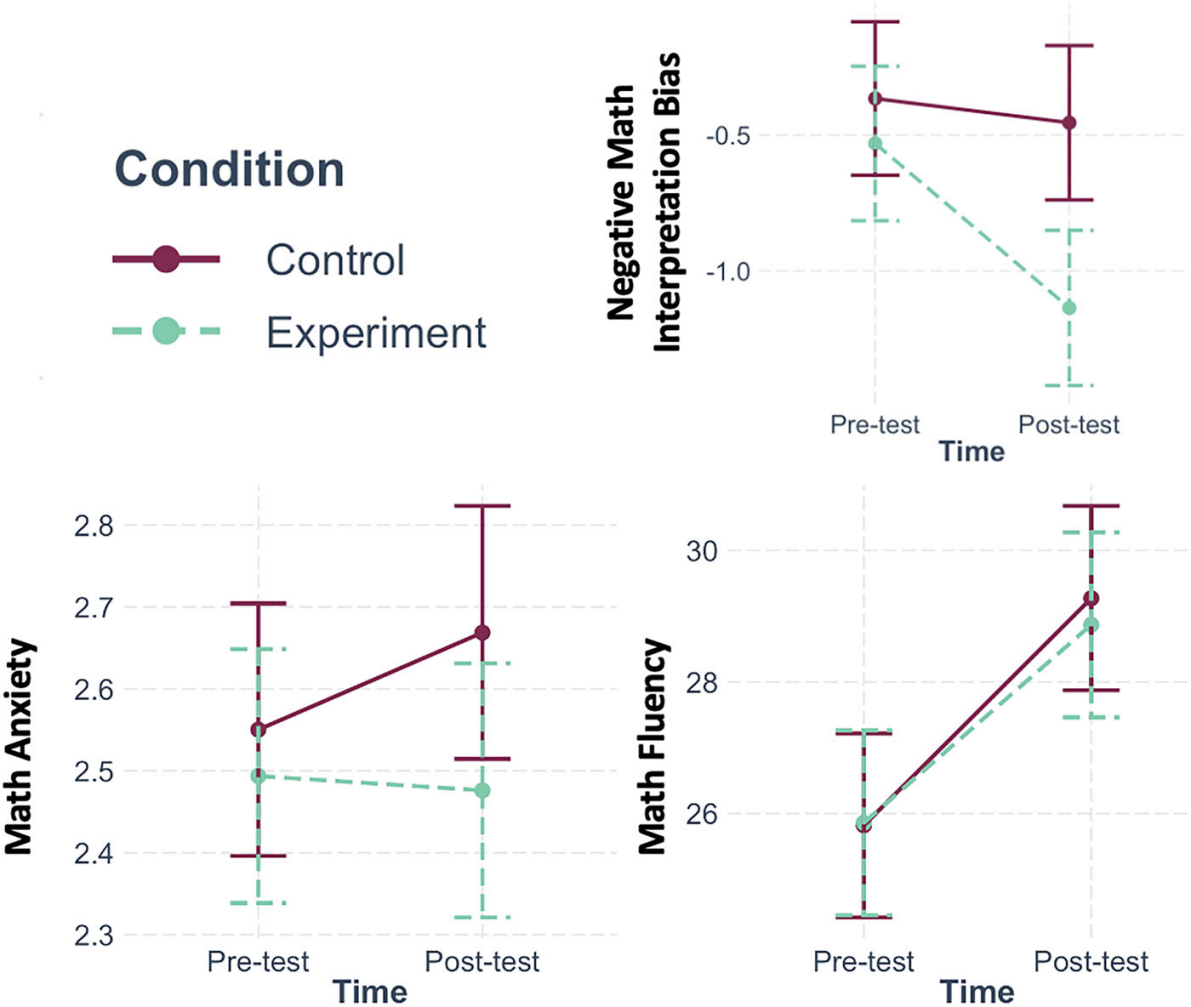
Time (Pre‐test vs. Post‐test) × Condition (Control vs. Experiment) interactions for negative mathematics interpretation bias, mathematics anxiety, and mathematics fluency.

To answer the first two sets of questions, we conducted and reported two complementary sets of the same analyses for both questions. To test hypotheses 1a and 2a, we analyzed multiple linear regression models predicting each of the post‐test study variables to test for differences by Condition. Condition (Experiment vs. Control) was included as the main predictor, and covariates of age and gender, as well as pre‐test variables of the same post‐test variable, were also included (Table 2). To test for differences in pre‐ and post‐test score changes by Condition as hypothesized in 1b and 2b, we conducted repeated measures analysis of covariance (ANCOVA) models for each variable (Table 3). In each analysis, we included a Time (Pre‐test vs. Post‐test assessment) × Condition (Experiment vs. Control) interaction, lower‐order terms of Time and Condition, and covariates of age and gender. Mean difference tests and estimated marginal means across Conditions and Time Points are presented in Table 4. Time × Condition interactions for each variable are visualized in Figure 2.

**TABLE 2:** Results from multiple linear regression models predicting post‐test variables.

Variables	Negative mathematics bias		Mathematics anxiety		Mathematics fluency	
	*B* (SE)	*β*	*B* (SE)	*β*	*B* (SE)	*β*
*Constant*	−0.20 (0.63)	—	0.01 (0.31)	—	8.41 (3.31)	—
Condition^a^	−0.52^**^ (0.11)	−0.18	−0.12^*^ (0.05)	−0.08	−0.32 (0.52)	−0.02
Pre‐test variable	0.98^**^ (0.04)	0.86	0.93^**^ (0.03)	0.91	0.74^**^ (0.04)	0.85
Age	−0.01 (0.03)	−0.02	0.01 (0.02)	0.03	0.07 (0.15)	0.02
Gender^b^	−0.02 (0.13)	−0.004	0.08 (0.06)	0.04	0.87 (0.64)	0.06
						
Adjusted *R* ^2^	0.78^**^		0.82^**^		0.74^**^	
*N*	176		178		178	

*Note*: *B*, unstandardized beta estimate; *β*, standardized beta estimate.

Abbreviation: SE, standard error.

^a^Control = 0, experiment = 1.

^b^Female = 0, male = 1.

^*^ *p* < 0.05; ^**^ *p* < 0.001.

**TABLE 3:** Repeated measures analysis results for study variables.

	Negative mathematics interpretation bias			Mathematics anxiety			Mathematics fluency		
**Source**	***F***	***p***	**ES (*η* ^2^)**	***F***	***p***	**ES (*η* ^2^)**	***F***	***p***	**ES (*η* ^2^)**
*Between‐subjects effects*									
Condition^a^	**4.41**	**0.04**	**0.03**	1.26	0.26	0.007	0.03	0.86	<0.001
Age	0.12	0.73	0.001	0.21	0.65	0.001	3.68	0.06	0.02
Gender^b^	**6.43**	**0.01**	**0.04**	**7.88**	**0.006**	**0.04**	**19.81**	**<0.001**	**0.10**
									
*Within‐subjects effects*			*Part η* ^2^			*Part η* ^2^			*Part η* ^2^
Time (Pre vs. Post)	0.01	0.92	<0.001	0.56	0.45	0.003	0.17	0.68	0.001
Time × Condition	**23.10**	**<0.001**	**0.12**	**5.36**	**0.02**	**0.03**	0.28	0.60	0.002
Time × Age	0.22	0.64	0.001	0.73	0.39	0.004	2.05	0.15	0.012
Time × Gender	0.001	0.98	<0.001	2.98	0.09	0.02	0.59	0.45	0.003

*Note*: **Bolded** = Estimates of predictors with significance levels of *p* < 0.05. *η* ^2^, eta squared.

Abbreviations: ES, effect size; *Part η* ^2^, partial eta squared.

^a^Control = 0, experiment = 1.

^b^Female = 0, male = 1.

**TABLE 4:** Estimated marginal means and mean difference tests across conditions and time points.

Variable	Condition	T1 (Pre‐test)	T2 (Post‐test)	*T1 versus T2^b^*
Negative mathematics bias	Control	−0.37	−0.45	*0.09 (0.08)*
	Experimental	−0.54	−1.14	0.60** (0.08)
	*Control versus experiment^a^*	*0.17 (0.20)*	0.69* (0.22)	
				
Mathematics anxiety	Control	2.56	2.66	−0.11* (0.04)
	Experimental	2.49	2.48	*0.01 (0.04)*
	*Control versus experiment^a^*	*0.07 (0.11)*	*0.18 (0.12)*	
				
Mathematics fluency	Control	25.85	29.24	−3.39** (0.41)
	Experimental	25.83	28.90	−3.08** (0.41)
	*Control versus experiment^a^*	*0.03 (1.10)*	*0.34 (0.97)*	

*Note*: Mean difference tests and standard errors reported in parentheses are italicized.

Abbreviations: T1, time 1; T2, time 2.

^a^Control condition is the reference group in analyses.

^b^Time 1 is the reference group in analyses.

* *p* < 0.01; ** *p* < 0.001.

#### 3.1.1 Research Question 1: CBM‐MI Near Transfer Effects

##### 3.1.1.1 Negative Mathematics Interpretation Bias

Condition was found to be a small and statistically significant predictor of post‐test negative mathematics interpretation bias (Table 2; *β* = −0.18, *p <* 0.001). Students in the Experiment condition reported lower post‐test negative interpretation bias than students in the Control condition. Pre‐test negative mathematics interpretation bias was also a strong, significant predictor of post‐test negative mathematics interpretation bias (*β* = 0.86, *p* < 0.001).

In the repeated measures ANCOVA model for mathematics interpretation, we found a moderate‐to‐strong and statistically significant Time × Condition interaction (Table 3; partial *η* ^2^ = 0.12) that indicated two patterns of pre‐to post‐test change (Table 4). First, the negative mathematics interpretation bias scores for students in the Experiment condition significantly decreased from pre‐test (EMM = −0.54) to post‐test (EMM = −1.14), but no significant changes were found for students in the Control condition. Second, the gap for negative mathematics interpretation bias between the Experiment and Control conditions was not different from each other at the pre‐test but was significantly different at the post‐test (*p* = 0.002).

In addition to the Time × Condition interaction, gender was also a significant predictor (Table 3). Male participants (EMM = −1.09) reported lower negative mathematics bias overall across both time points and collapsed across conditions than female participants (EMM = −0.49).

#### 3.1.2 Research Question 2: CBM‐MI Far Transfer Effects

##### 3.1.2.1 Mathematics Anxiety

Condition was found to be a very small but statistically significant predictor of post‐test mathematics anxiety (Table 2; *β* = −0.08, *p* = 0.016) in the regression model. Students in the Experiment condition reported lower post‐test mathematics anxiety than students in the Control condition. Pre‐test negative mathematics anxiety was also a strong and significant predictor of post‐test negative mathematics anxiety (Table 2; *β* = 0.91, *p* < 0.001).

A moderately small and statistically significant Time × Condition interaction was found in the repeated measures ANCOVA model for mathematics anxiety (Table 3; partial *η* ^2^ = 0.03). As shown in Table 4, this interaction indicated that students in the Control condition significantly increased their mathematics anxiety levels from pre‐test (EMM = 2.56) to post‐test (EMM = 2.66), whereas students in the Experiment condition did not have significantly different levels of mathematics anxiety across time points (pre‐test EMM = 2.49; post‐test EMM = 2.48).

In addition to the Time × Condition interaction, gender was also a small‐to‐moderately sized significant predictor (Table 3; partial *η* ^2^ = 0.04). Male participants reported lower mathematics anxiety overall across both time points and collapsed across conditions (EMM = 2.26) than female participants (EMM = 2.63).

##### 3.1.2.2 Mathematics Achievement

In the regression model, only pre‐test mathematics fluency was a strong, significant predictor of post‐test mathematics fluency (Table 2; *β* = 0.85, *p* < 0.001). There was also no significant Time × Condition interaction effect found on mathematics fluency in the repeated measures ANCOVA model (Table 3). However, statistically significantly higher scores were found for male participants (EMM = 31.50) overall across both time points and collapsed across conditions than female participants (EMM = 26.29).

### 3.2 Research Question 3: Negative Mathematics Interpretation Bias as a Mediator

To test our third research question regarding the mediating role of negative mathematics interpretation bias, we conducted separate mediation analyses with mathematics anxiety and mathematics fluency scores as dependent variables. Despite the lack of a significant direct relation between Study Condition and mathematics fluency (Table 3), it is still possible for Study Condition to have an indirect relation through a mediator [66]. Study Condition was included as the Predictor variable, and Mathematics interpretation bias was included as the Mediator variable. The same covariates described for Research Questions 1 and 2 were also included. We used the PROCESS macro (model 4) with 10,000 bootstrap samples and 95% bias‐corrected confidence intervals [67]. Table 5 presents paths b, c′, and indirect/total effect results from the mediation models (Table 3 presents results for paths a and c).

**TABLE 5:** Mediation analysis results with mathematics interpretation bias as the mediator.

	Mathematics anxiety (Y)			Mathematics fluency (Y)		
Source	B (SE)	*β*	*t*	B (SE)	*β*	*t*
**Models with M as predictor (path b)**						
*Constant*	0.25 (0.28)		0.90	7.92 (3.16)		2.51*
Post‐test negative mathematics bias (M)	0.11 (0.02)	0.22	6.03**	−0.17 (0.17)	−0.04	−0.96
Pre‐test of DV	0.80 (0.04)	0.78	20.94**	0.76 (0.04)	0.85	21.01**
Age	0.02 (0.01)	0.04	1.45	0.06 (0.14)	0.02	0.44
Gender^a^	0.10 (0.06)	0.06	1.85	0.77 (0.64)	0.05	1.20
**Models with both X and M as predictors (path c′)**						
*Constant*	0.32 (0.29)		1.13	8.52 (3.29)		2.59*
Condition^b^ (X)	−0.05 (0.05)	−0.07	−1.05	−0.35 (0.53)	−0.05	−0.66
Post‐test negative mathematics bias (M)	0.11 (0.02)	0.21	5.58**	−0.19 (0.18)	−0.04	−1.08
Pre‐test of DV	0.80 (0.04)	0.79	20.92**	0.76 (0.04)	0.85	20.96**
Age	0.02 (0.01)	0.04	1.19	0.04 (0.15)	0.01	0.29
Gender^a^	0.09 (0.06)	0.05	1.68	0.70 (0.65)	0.04	1.09
						
	**Effect (SE)**	**LL**	**UL**	**Effect (SE)**	**LL**	**UL**
Indirect effect of X on Y (a × b)	−0.06 (0.02)	−0.09	−0.03	0.10 (0.08)	−0.05	0.29
Total effect of X on Y	−0.11 (0.05)	−0.20	−0.02	−0.25 (0.47)	−1.17	0.67

*Note*: Model result with Y as the predictor variable and M as the dependent variable is presented in Table 2. 10,000 bootstrapped samples were used to calculate 95% confidence interval estimates. B, unstandardized coefficient; β, standardized coefficient.

Abbreviations: DV, outcome variable in model; LL, lower level 95% confidence interval; SE, standard error; UL, upper level 95% confidence interval.

^a^Female = 0, male = 1.

^b^Control = 0, experiment = 1.

*p < 0.05; **p < 0.001.

**FIGURE 3:**
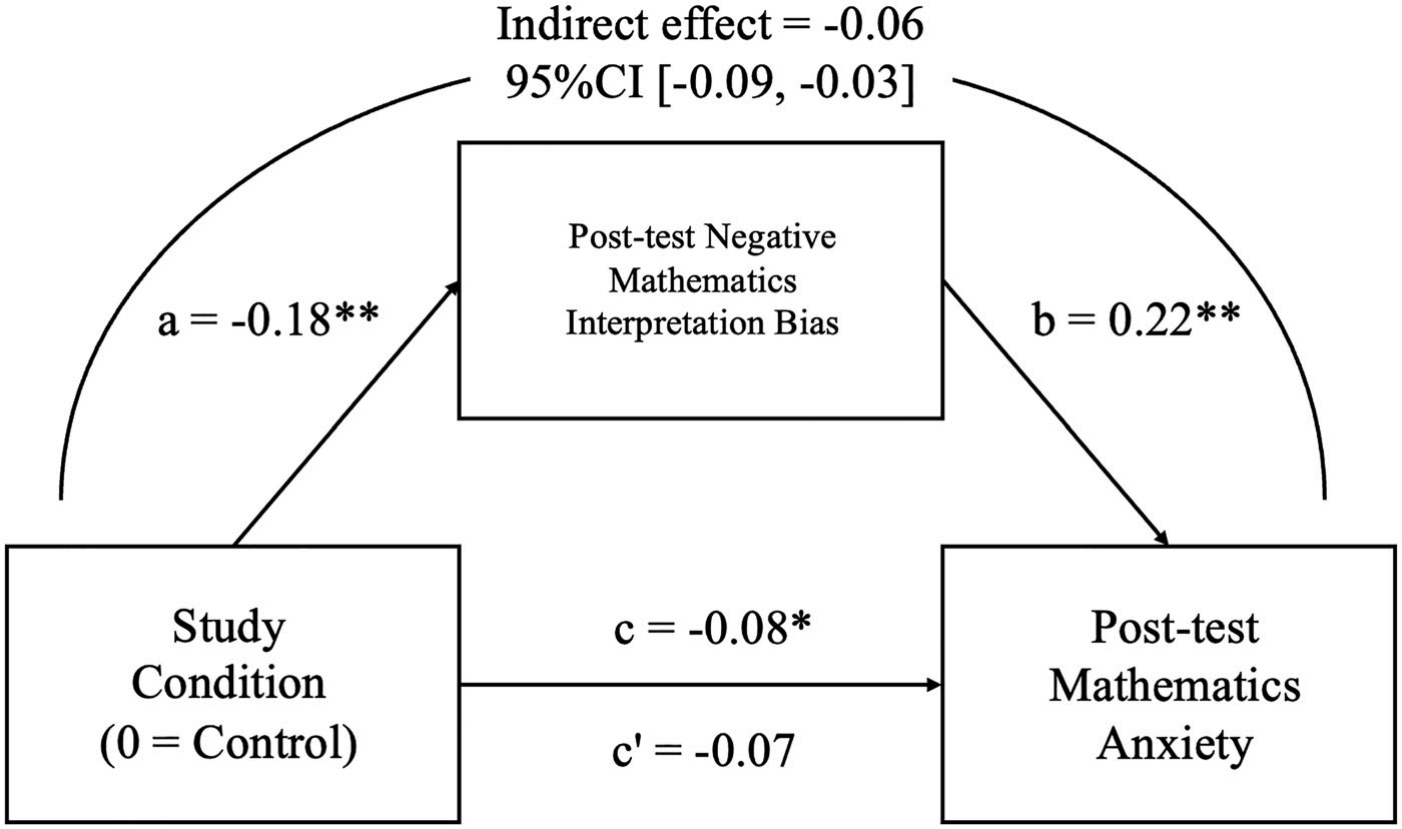
Standardized regression coefficients (*β*) for main mediation model paths with Study Condition predicting mathematics anxiety through negative mathematics interpretation bias (*p < 0.05; **p < 0.001).

For the mediation model predicting mathematics anxiety (Figure 3), path b indicated that the post‐test negative mathematics interpretation bias was a small but significant predictor of mathematics anxiety. The direct effect of Study Condition was very small but significant before accounting for interpretation bias (path c) but no longer significant after accounting for interpretation bias (path c′). The indirect effect of Study Condition on mathematics anxiety through negative mathematics interpretation bias was significant (a × b = −0.06, SE = 0.02, 95% CI [−0.09, −0.03]), indicating full mediation.

For the mediation model predicting mathematics fluency, path b indicated that the post‐test negative mathematics interpretation bias did not significantly predict fluency. The direct effect of Study Condition on fluency was nonsignificant both before (path c) and after accounting for interpretation bias (path c′), and the indirect effect of Study Condition on fluency through negative mathematics interpretation bias was also nonsignificant (a × b = 0.10, SE = 0.08, 95% CI [−0.05, 0.29]).

## 4 Discussion

In the present study, our goal was to understand if principles from cognitive bias modification for interpretation bias could generalize to a domain‐specific mathematics context (CBM‐MI) and produce near transfer effects on negative mathematics interpretation bias. Additionally, we sought to understand if this paradigm could have far transfer effects on mathematics anxiety and mathematics achievement, and if negative mathematics interpretation bias could mediate the intervention effect. Overall, our study results suggest that CBM‐I is generalizable to mathematics contexts and, compared to an active nonmathematics control condition, can produce near transfer effects on negative mathematics interpretation bias. We also provide initial evidence for the potential use of mathematics‐specific CBM‐I paradigms in college students compared to an active control condition to buffer against increases in mathematics anxiety through lower negative mathematics interpretation bias [56]. Our study has implications for theory on negative interpretations of mathematics scenarios and mathematics anxiety as well as practical applications for mathematics anxiety interventions in mathematics contexts.

### 4.1 Near Transfer Effects of CBM‐MI

Past research on CBM‐I indicates its effectiveness in significantly reducing negative and threat‐oriented biased interpretations of general and social situations/stimuli [40, 58]. In testing our first research question regarding whether CBM‐MI has significant effects on mathematics‐specific interpretation bias, we found support in favor of our hypothesis that it did: specifically, participants in the CBM‐MI condition reported lower levels of post‐test negative mathematics interpretations bias compared to participants in the active nonmathematics control condition. We also found a significant and moderately sized difference in the pre‐ to post‐test negative mathematics interpretations bias changes for participants in the CBM‐MI condition, but not for the control condition. These findings align with past work on the effectiveness of CBM‐I on the beliefs it targets [47, 50] and suggest that training to resolve ambiguous mathematics‐related scenarios in a positive manner, compared to resolving nonmathematics scenarios in a neutral manner, is better at modifying interpretations of ambiguous mathematics scenarios to be less biased toward threatening interpretations.

As Ramirez et al. proposed in the interpretation account of mathematics anxiety development, students make meanings out of their past mathematics experiences which then become the interpretive lens they use to understand their present and future mathematics experiences [12]. Our study results suggest that implementing a formalized cognitive interpretation bias intervention paradigm using basic association principles to link positive resolutions with mathematics scenarios can reshape students’ interpretations to be less negatively biased. Thus, CBM‐MI has the potential to be used in educational settings to help change negative interpretations students might have developed about mathematics situations. For example, CBM‐MI can be used as a supplemental intervention or integrated into mathematics lessons to help reshape students’ interpretations about mathematics to be more adaptive. However, more research is needed to understand whether its implementation in real‐life instructional settings would be as effective at modifying negative interpretations as implementing the intervention in a controlled lab setting.

### 4.2 Far Transfer Effects of CBM‐MI and Negative Mathematics Bias as a Mediator

Given significant impacts of CBM‐I on outcomes beyond cognitive interpretation bias, such as anxiety [40, 58] and the relation between mathematics anxiety and mathematics achievement [6], we hypothesized significant far transfer effects and greater pre‐ to post‐test changes of CBM‐MI on both mathematics anxiety and mathematics achievement in the Experiment condition than the Control condition. We also hypothesized that negative mathematics interpretation bias would mediate the relation between CBM‐MI and both mathematics anxiety and mathematics achievement. As discussed in the next few paragraphs, our results for our second and third research questions only partially supported our hypotheses.

#### 4.2.1 Mathematics Anxiety

We found that students in the CBM‐MI condition had statistically significant lower reports of post‐test mathematics anxiety than students in the Control condition, although the effect size indicated that this difference between groups was very small. In contrast to our hypothesis of mathematics anxiety *reductions* in the CBM‐MI group, we found a significant *increase* in mathematics anxiety for the control group and only a slight *decrease* for the experiment group. The pre‐ to post‐test change in mathematics anxiety was significantly different by Condition, but the effect size of the difference in change over time between groups was also small. These findings suggest at least two possibilities. First, it could be that reading through ambiguous scenarios related to grocery shopping, visiting the park, and a dinner outing, as the students in the Control condition did, has a small but important activation on college students’ mathematics anxiety [68, 69]. However, Control condition participants were tasked with resolving scenarios in a neutral manner, and as such, we would still not have expected increases in mathematics anxiety to occur given the neutral resolution of the ambiguous scenarios. Second, it is possible that practicing resolutions of ambiguous mathematics scenarios in a positive manner, as students in the CBM‐MI condition did, protected students from experiencing an increase in mathematics anxiety, akin to a reduced sensitization response with frequent exposure to stressful mathematics scenarios and stimuli [70]. This also aligns with past research that has suggested that more frequent engagement with mathematics resources could habituate students to their mathematics anxiety [71].

In addition to the small direct effects of CBM‐MI on mathematics anxiety, we also found that post‐test negative mathematics interpretation bias, accounting for pre‐test levels, mediated the relation between Study Condition and post‐test mathematics anxiety. This supports past research finding similar mediating effects of negatively biased interpretation processing between CBM‐I and anxiety [56]. Specifically, we found that students in the Control condition had higher levels of post‐test negative mathematics interpretation bias than students in the CBM‐MI condition, which predicted higher post‐test levels of mathematics anxiety. These findings suggest that differences in negative mathematics interpretation bias after engaging in either CBM‐MI or an active nonmathematics control condition may serve as a mechanism that influences students’ mathematics anxiety. Conversely, increases in anxiety may be buffered for the CBM‐MI group due to lower levels of negative mathematics interpretation bias. Overall though, more empirical research is needed to better understand if either of these two explanations have significant merit in explaining why significant, yet small, changes in mathematics anxiety were found between the two conditions in our study.

#### 4.2.2 Mathematics Achievement

We did not find any CBM‐MI impacts or pre‐ to post‐test changes on our mathematics achievement indicator of mathematics fluency. Regarding our mediation analyses, we also did not find that negative mathematics interpretation bias indirectly influenced the link between CBM‐MI and mathematics fluency. These findings provide evidence of little, if any, effects of CBM‐MI on mathematics fluency, which aligns with some research showing limited effects of CBM‐I on secondary symptoms beyond those primary symptoms of anxiety or biases that are targeted by the intervention [53]. Importantly, our simple measure of mathematics achievement through fluency on a whole number multiplication quiz may not have been optimal in manipulating working memory for our college‐aged student sample. Working memory is theorized to be an important cognitive resource that is depleted when mathematics anxiety is elevated that can negatively influence mathematics task performance [72−74]. Related to our study, it may be that CBM‐MI effects could differ in scale if mathematics achievement is measured with a test assessing more advanced or complex mathematics knowledge. The amount of working memory resources used during a mathematics test can differ depending on the mathematics skill being assessed [75]. As such, more research is needed to investigate the specific types of mathematics performance that negative mathematics interpretation bias might interfere with and if CBM‐MI can influence performance on those mathematics tasks.

### 4.3 Limitations and Future Directions

The present study and its findings do have important limitations that should be noted. First, our findings are relevant to college students who were not selected for high levels of mathematics anxiety. Interventions like CBM‐MI may have different effects on younger students, nonstudent adults, or samples selected for high levels of mathematics anxiety. Second, we did not test for long‐term effects on negative mathematics bias or anxiety [76], the effectiveness of CBM‐MI to that of other mathematics anxiety interventions [77], or isolated effects of CBM‐MI on different types of anxiety (i.e., general anxiety or test anxiety). Future research should be done to explore the benefits and long‐term effects of CBM‐MI on underlying cognitive processes, as well as compare the effects of other interventions to that of CBM‐MI and whether the effects are domain‐specific to mathematics anxiety.

Third, our study design is also limited. As mentioned in the previous section, our multiplication quiz may have been too simple for the college‐aged students in our sample. Furthermore, our initial score calculation for mathematics achievement included an accuracy percentage that unfortunately had a ceiling effect, and thus we excluded this score from our analyses due to its restricted variance. Future studies should evaluate the impacts of CBM‐MI either on different mathematics tests assessing advanced or complex mathematics skills that more directly manipulate working memory or a more difficult version of a multiplication task that captures more variability in accuracy. Our mathematics anxiety scale also assessed trait mathematics anxiety, which by definition is considered a durable personality trait, as opposed to state emotions, which are in‐the‐moment emotions that fluctuate depending on the context [78]. Assessments of state mathematics anxiety, including physiological measures such as heart rate or skin conductance, may be more malleable than trait anxiety and more sensitive to CBM‐MI. Additionally, we employed an active control condition that did not include specific mathematics learning, evaluation, or classroom situations. Thus, the effects we found are specific to these conditions and, as such, may have been due to the difference in presentation of mathematics content between the two conditions. For example, it is possible that a general interpretation bias condition may also buffer against increases in mathematics anxiety compared to an active control without mathematics. The implementation of a no‐control situation, a placebo control condition with mathematics involved, or a different active control condition where mathematics is involved but a nonpositive bias is targeted (e.g., negative bias or neutral bias training) should be tested in future research to address the mathematics‐specific nature of this causal relation between CBM‐MI and mathematics bias and anxiety [79]. Furthermore, our baseline assessments indicated that despite random assignment to condition, there were significant differences in students’ age across groups. Although age did not influence our dependent variables, pre‐test differences with random assignment can be a concern, as it suggests the potential of unobserved variables that may have influenced the impact of the intervention [80, 81]. Future experimental research should carefully consider covariate pre‐testing or matching groups to evenly balance out and account for relevant characteristics to the outcome variables.

Fourth, there is room for improvement in the features and logistics of the CBM‐MI we developed. The intervention itself was brief and done in a single, hour‐long session in an online setting. Past work on interpretation bias and mathematics anxiety interventions suggests that multiple sessions are better than one session [82], with some of the strongest effects found for interventions that are 3 weeks or longer [77]. The use of a single session in the present study may have limited our findings. Additionally, adults may have found the scenario‐based task boring [83], but improvements such as personalization of mathematics content based on popular culture references can be useful characteristics of interventions to modify and increase college student interest and mathematics learning [84]. The present study's scenario conditions did not include personalization or popular culture references and instead were standardized across participants with little specificity regarding popular culture from any time period.

Finally, there are some analytic limitations in this study to acknowledge. Although we tested for a causal relation in an experimental design that used random assignment, the effects we did find on mathematics anxiety were very small to moderate. Additionally, post‐hoc power analyses indicate that we are underpowered for the simple mediation analysis we conducted given the sizes of the a, b, and c′ paths and sample size we had for this analysis [85, 86]. Finally, additional mediational pathways may exist that we did not test for due to our sample size. For example, it is possible that the reductions in anxiety freed up cognitive resources that allowed for an improvement in mathematics task performance. A sequential mediation analysis could be tested with both interpretation bias and mathematics anxiety as mediators in the relation between CBM‐MI and mathematics achievement, but we are quite underpowered in the present study to provide reliable results for these complex analyses with our current sample size. Future research should consider the interplay between mathematics anxiety and performance when testing for changes in negative interpretation bias to further test different theoretical models such as the cognitive interference model explaining the link between mathematics achievement and anxiety [35−37].

## 5 Conclusion

The present study adds to the limited research investigating interpretation bias in a mathematics context and supports past research finding strong links between mathematics anxiety and mathematics interpretation bias. Our study also provides support for the use of a brief CBM‐MI in producing near transfer effects in reducing negative interpretation bias toward mathematics contexts. There is also initial evidence of far transfer CBM‐MI effects on certain dimensions of mathematics anxiety and achievement scores. Overall, the present study's findings on this brief CBM‐MI are promising and set up the potential for future work to continue investigating CBM‐MI as a new addition to the lineup of interventions that can be used to reduce mathematics anxiety in college students.

## Author Contributions

C.B. designed the study, acquired data, and conducted data analysis. C.B. and X.Y. prepared original versions of the draft. C.B., X.Y., and Z.W. interpreted results and provided critical edits and review of the final submitted manuscript.

## Conflicts of Interest

The authors declare no potential conflicts or competing interests with respect to the research, authorship, and/or publication of this article.

## IRB Statement

This study received ethical approval from the Texas A&M University Institutional Review Board (approval #IRB2021‐0244M), and participants provided consent to participate in the study.

## Supporting information




**Supplementary Material**: nyas70405‐sup‐0001‐TableS1‐S9.docx

## Data Availability

Materials and code for reproducing all analyses are open and available online via the Open Science Framework (https://osf.io/8t7zh/).
