# Supplementary material for: Initial Evidence of Mathematics Interpretation Bias Intervention Effects on College Students’ Mathematics Anxiety
Source: Ann N Y Acad Sci. 2026 Sep 24;1563(1):e70405. doi: 10.1111/nyas.70405 (PMC13602885; doi:10.1111/nyas.70405)
Supplement: Supplementary file 1 [file NYAS-1563-0-s001.docx]

**Initial Evidence of the Effects of a Mathematics Interpretation Bias Intervention on**

**College Students’ Mathematics Anxiety**

**Supplemental Materials**

**Table of Contents**

*Missing Data and Descriptive Statistics* ………………..…………………….................. PAGE 2

*Supplemental Table S1*…………………………………………………………………… PAGE 3

*Supplemental Table S2*…………………………………………………………………… PAGE 4

*Supplemental Table S3*…………………………………………………………………… PAGE 5

*Supplemental Table S4*…………………………………………………………………… PAGE 5

*Supplemental Table S5*…………………………………………………………………… PAGE 6

*Supplemental Table S6*…………………………………………………………………… PAGE 7

*Supplemental Table S7*…………………………………………………………………… PAGE 8

*Supplemental Table S8*…………………………………………………………………… PAGE 9

**Missing Data and Descriptive Statistics**

One student had missing data for one item on the 9-item post-test mathematics anxiety scale. Seven students were missing data for one item on the 24-item pre-test negative mathematics interpretation bias scale, 1 student was missing data on 2 items, and 1 student was missing data on 6 items. One student was missing data on one item on the post-test negative mathematics interpretation bias scale. Two students did not complete any items on the post-test negative mathematics interpretation bias scale due to technical glitches in the experiment survey for the post-test interpretation bias assessment. Average scores were calculated based on available data.

Levene’s test indicated equality of variances for the Experiment and Control conditions across all study variables except for pre-test mathematics anxiety (Levene’s test = 3.94, *p* = .049) and pre-test WSAP-M negative trials average rating (Levene’s test = 5.82, *p* = .017). Therefore, Welch’s test was used in place of one-way ANOVA to evaluate significant differences across groups in the mathematics anxiety and WSAP-M negative trial variables (both reported in supplemental materials).

| **Supplemental Table S1**  *Student Reported Undergraduate Degree Majors* |  |  |
| --- | --- | --- |
| Major | Frequency | Percentage |
| Engineering | 48 | 26.5 |
| Business and Economics (Marketing, Business, Business Administration, Economics, Management Information Systems, Supply Chain Management) | 20 | 11.0 |
| Psychology and Neuroscience | 17 | 9.4 |
| Sciences (Physics, Chemistry, Biochemistry, Biology, Microbiology, Genetics) | 14 | 7.7 |
| Biomedical Sciences | 13 | 7.2 |
| Public Health | 9 | 5.0 |
| Animal Sciences and Zoology | 7 | 3.9 |
| Exercise Science, Kinesiology, Food Science, Nutrition | 6 | 3.3 |
| Government and Public Service | 6 | 3.3 |
| Education and Human Development | 5 | 2.8 |
| Computer Science | 5 | 2.8 |
| Communications | 4 | 2.2 |
| University or General Studies | 4 | 2.2 |
| Applied Biological Sciences (Environmental Studies, Horticulture, Environmental Geoscience, Bioenvironmental Sciences) | 4 | 2.2 |
| Recreation, Parks, and Tourism Sciences | 3 | 1.7 |
| Forensic and Investigative Sciences | 3 | 1.7 |
| Statistics and Applied Mathematics | 2 | 1.1 |
| English | 2 | 1.1 |
| Sociology | 2 | 1.1 |
| Dual/double majors | 2 | 1.1 |
| History | 1 | 0.6 |
| Visualization and Performance | 1 | 0.6 |
| Anthropology | 1 | 0.6 |
| Oceanography | 1 | 0.6 |
| Architecture or Environmental Design | 1 | 0.6 |

| **Supplemental Table S2**  *One-Way Analysis of Variance Test by Condition for Pre-test Study Variables* | | | | |
| --- | --- | --- | --- | --- |
| Variables | Experiment (N = 89) | Control  (N = 92) | One-way ANOVA |  |
| Negative Mathematics Interpretation Bias | -0.50 | -0.36 | 0.51 (1, 179) |  |
| Mathematics Anxiety | 2.50 | 2.55 | W: 0.18 (1, 170.26) |  |
| Mathematics Fluency | 25.83 | 25.97 | 0.02 (1, 179) |  |
| Age (Years) | 19.67 | 20.43 | 8.58 (1, 179)* |  |
| Gender^1^ | - | - | 1.95 (1, 177) |  |
| *Note*. ^1^Female = 0, Male = 1; W= Welch’s test; KWH: Kruskal-Wallis H test; **p* < 0.01. | | | |  |

| **Supplemental Table S3**  *Pearson’s Correlation Coefficients Between Pre-test Study Variables* | | | | | | |
| --- | --- | --- | --- | --- | --- | --- |
| Variables | Negative Bias | Anxiety | Fluency | Age | *Positive Trials* |  |
| Negative Mathematics Interpretation Bias | 1.0 |  |  |  |  |  |
| Mathematics Anxiety | .68** | 1.0 |  |  |  |  |
| Mathematics Fluency | -.03 | -.08 | 1.0 |  |  |  |
| Age (Years) | .02 | .06 | -.18* | 1.0 |  |  |
| Gender^1,2^ | -.19* | -.22** | .32** | -.13 | 1.0 |  |
| *Positive Mathematics Interpretations* | -.78** | -.40** | .05 | -.04 | .18* |  |
| *Negative Mathematics Interpretations* | -.88** | .69** | -.01 | <.001 | -.15* |  |
| *Note*. **p* < 0.05; ***p* < 0.01; ^1^Spearman's *rho* correlations reported due to categorical variable; ^2^Female = 0, Male = 1; Eval = Evaluation; *N* = 181 except for Gender correlations where *N* = 179; Variables only reported in Supplemental Materials are *italicized*. | | | | | | |

| **Supplemental Table S4**    *Descriptive Statistics for Pre-Test Study Negative and Positive Math Interpretation Scores* | | | | | | | | | |  |
| --- | --- | --- | --- | --- | --- | --- | --- | --- | --- | --- |
| Variables | N | Actual  Min-Max | Mean | SD | Skew | Kurt | Experiment (N = 89) | Control (N = 92) | One-way ANOVA | |
| Positive Mathematics Interpretations | 181 | 1.00 to 5.75 | 4.07 | 0.67 | -0.62 | 1.75 | 4.11 | 4.02 | 0.74 (1, 179) | |
| Negative Mathematics Interpretations | 181 | 1.00 to 5.75 | 3.64 | 0.88 | -0.10 | 0.21 | 3.61 | 3.66 | W: 0.16 (1, 164.45) | |
| *Note*. ^1^Female = 0, Male = 1; SD = Standard Deviation; Skew = Skewness; Kurt = Kurtosis; W: Welch’s test. | | | | | | | | | |  |

| **Supplemental Table S5**  *Linear Regression Analysis Results Predicting Post-test Negative and Positive Mathematics Interpretation Scores* | | | | |
| --- | --- | --- | --- | --- |
| Variables | **Negative Mathematics Interpretations** | | **Positive Mathematics Interpretations** | |
|  | ***B* (SE)** | ***β*** | ***B* (SE)** | ***β*** |
| *Constant* | .69(.55) |  | .74(.48) |  |
| Condition^1^ | -0.23*(.09) | -.12 | 0.31**(.07) | .21 |
| Pre-test variable | 0.86**(.05) | .79 | 0.80**(.05) | .73 |
| Age | -0.01 (.03) | -.02 | .003 (.02) | .01 |
| Gender^2^ | -0.13 (.10) | -.06 | -0.03 (.09) | -.02 |
|  |  |  |  |  |
| Adjusted *R^2^* | .65** | | .58** | |
| *N* | 176 | | 176 | |
| *Note*. ^1^Control=0, Experiment = 1; ^2^Female = 0, Male = 1; ***p* < 0.01, ***p* < 0.001; SE = Standard Errors | | | | |

| **Supplemental Table S6**  *Repeated Measures Analysis of Covariance Results for Positive and Negative Mathematics Interpretation Scores* | | | | | | |
| --- | --- | --- | --- | --- | --- | --- |
|  | **Negative Mathematics Interpretations** | | | **Positive Mathematics Interpretations** | | |
| **Source** | ***F*** | ***p*** | **ES** | ***F*** | ***p*** | **ES** |
| *Between-subjects effects* | |  | *η^2^* |  |  | *η^2^* |
| Condition^1^ | 1.77 | .19 | 0.01 | **6.25** | **.03** | **0.03** |
| Age | 0.30 | .59 | 0.002 | <0.001 | .99 | <0.00 |
| Gender^2^ | **5.03** | **.03** | **0.03** | **4.70** | **.03** | **0.03** |
|  |  |  |  |  |  |  |
| *Within-subjects effects* | |  | *Partial η^2^* |  |  | *Partial η^2^* |
| Time (Pre vs. Post) | 0.002 | .96 | <0.001 | 0.04 | .85 | <0.001 |
| Time x Condition | **6.31** | **.01** | **0.035** | **14.55** | **<.001** | **0.08** |
| Time x Age | 0.17 | .68 | 0.001 | 0.03 | .86 | <0.001 |
| Time x Gender | 0.72 | .40 | 0.004 | 0.92 | .34 | 0.005 |
|  |  |  |  |  |  |  |
| *Notes*. **Bolded** = Predictors with significance levels of *p* < .05**;** ^1^Control = 0, Experiment = 1; ^2^Female = 0, Male = 1; ES = Effect size; *partial* *η*^2^ = partial eta squared. | | | | | | |

| **Supplemental Table S7**  *Estimated Marginal Means and Mean Difference Tests Across Conditions and Time Points for Positive and Negative Mathematics Interpretations* | | | | |
| --- | --- | --- | --- | --- |
| **Variable** | **Condition** | **T1 (Pre-test)** | **T2 (Post-test)** | *T1 v T2^a^* |
| Negative mathematics interpretations | Control | 3.65 | 3.56 | *.09 (.06)* |
|  | Experimental | 3.59 | 3.27 | *-.32*** (.06)* |
|  | *Control v Experiment^a^* | *.07 (.14)* | *.29* (.15)* |  |
| Positive mathematics interpretations | Control | 4.02 | 4.01 | *.01 (.05)* |
|  | Experimental | 4.12 | 4.41 | *-.29*** (.05)* |
|  | *Control v Experiment^a^* | *-.10 (.10)* | *-.40*** (.11)* |  |
| *Notes.* Mean Difference tests and standard errors reported in parentheses are *italicized*; *p < .05; ** p < .01; *** p < .001; ^a^Time 1 and Control condition are Reference groups in analyses. | | | | |
|  | | | | |

| **Supplemental Table S8**  *Mediation Analysis Results with Positive Mathematics Interpretation Trials as the Mediator* | | | | | | | |
| --- | --- | --- | --- | --- | --- | --- | --- |
|  | **Mathematics Anxiety (Y)** | | | **Mathematics Fluency (Y)** | | |  |
| **Source** | **B(SE)** | ***β*** | **t** | **B(SE)** | ***β*** | **t** |  |
| **Models with M as Predictor (Path b)** |  |  |  |  |  |  |  |
| *Constant* | 0.53(.34) |  | 1.55 | 6.50(3.52) |  | 1.85 |  |
| Post-test Negative Mathematics Bias (M) | -0.13(.04) | -.13 | -3.71** | 0.34(.34) | .04 | 1.00 |  |
| Pre-test of DV | 0.89(.04) | .87 | 25.23** | 0.76(.04) | .85 | 21.01** |  |
| Age | 0.02(.01) | .04 | 1.19 | 0.07(.14) | .02 | 0.48 |  |
| Gender^2^ | 0.10(.06) | .06 | 1.71 | 0.80(.64) | .05 | 1.26 |  |
| **Models with Both X and M as Predictors (Path c')** |  |  |  |  |  |  |  |
| *Constant* | 0.59(.34) |  | 1.71 | 6.87(3.56) |  | 1.93 |  |
| Condition^1^ (X) | -0.07(.05) | -.10 | -1.46 | -0.38(.54) | -.06 | -0.72 |  |
| Post-test Negative Mathematics Bias (M) | -0.11(.04) | -.11 | -3.21* | 0.41(.35) | .05 | 1.15 |  |
| Pre-test of DV | 0.89(.04) | .87 | 25.35** | 0.76(.04) | .85 | 20.96** |  |
| Age | 0.01(.01) | .03 | 0.87 | 0.05(.15) | .01 | 0.32 |  |
| Gender^2^ | 0.09(.06) | .05 | 1.49 | 0.73(.64) | .05 | 1.14 |  |
|  |  |  |  |  |  |  |  |
|  | **Effect(SE)** | **LL** | **UL** | **Effect(SE)** | **LL** | **UL** |  |
| Indirect effect of X on Y (a x b) | -.04(.01) | -.07 | -.01 | .13(.11) | -.07 | .37 |  |
| Total effect of X on Y | -.11(.05) | -.21 | -.01 | -.26(.48) | -1.19 | .70 |  |
| *Notes*. Model result with Y as predictor variable and M as dependent variable is presented in Table 2; **p* < .05; ***p* < .001; B = unstandardized coefficient; SE = standard error; *β* = standardized coefficient; DV = outcome variable in model; ^1^Control = 0, Experiment = 1; ^2^Female = 0, Male = 1; LL = Lower Level 95% Confidence Interval; UL = Upper Level 95% Confidence Interval; 10,000 bootstrapped samples used to calculate 95% confidence interval estimates. | | | | | | | |

| **Supplemental Table S9**  *Mediation Analysis Results with Negative Mathematics Interpretation Trials as the Mediator* | | | | | | | |
| --- | --- | --- | --- | --- | --- | --- | --- |
|  | **Mathematics Anxiety (Y)** | | | **Mathematics Fluency (Y)** | | |  |
| **Source** | **B(SE)** | ***β*** | **t** | **B(SE)** | ***β*** | **t** |  |
| **Models with M as Predictor (Path b)** |  |  |  |  |  |  |  |
| *Constant* | -0.50(.27) |  | -1.82 | 8.76(3.31) |  | 2.64* |  |
| Post-test Negative Mathematics Bias (M) | -0.19(.03) | .23 | 6.05** | -0.19(.27) | -.03 | -0.70 |  |
| Pre-test of DV | 0.78(.04) | .77 | 19.66** | 0.76(.04) | .85 | 20.99** |  |
| Age | 0.02(.01) | .05 | 1.71 | 0.06(.14) | .02 | 0.41 |  |
| Gender^2^ | 0.10(.06) | .06 | 1.86 | 0.78(.64) | .05 | 0.22 |  |
| **Models with Both X and M as Predictors (Path c')** |  |  |  |  |  |  |  |
| *Constant* | -0.35(.29) |  | -1.21 | 9.33(3.49) |  | 2.68* |  |
| Condition^1^ (X) | -0.08(.05) | -.10 | -1.61 | -0.28(.52) | -.04 | -0.54 |  |
| Post-test Negative Mathematics Bias (M) | 0.18(.04) | .22 | 5.75** | -0.21(.27) | -.03 | -0.77 |  |
| Pre-test of DV | 0.79(.04) | .77 | 19.81** | 0.76(.04) | .85 | 20.93** |  |
| Age | 0.02(.01) | .04 | 1.32 | 0.04(.15) | .01 | 0.29 |  |
| Gender^2^ | 0.09(.04) | .05 | 1.63 | 0.73(.65) | .05 | 1.13 |  |
|  |  |  |  |  |  |  |  |
|  | **Effect(SE)** | **LL** | **UL** | **Effect(SE)** | **LL** | **UL** |  |
| Indirect effect of X on Y (a x b) | -.04(.02) | -.08 | -.01 | .05(.06) | -.06 | .19 |  |
| Total effect of X on Y | -.12(.05) | -.21 | -.02 | -.23(.48) | -1.19 | .69 |  |
| *Notes*. Model result with Y as predictor variable and M as dependent variable is presented in Table 2; **p* < .05; ***p* < .001; B = unstandardized coefficient; SE = standard error; *β* = standardized coefficient; DV = outcome variable in model; ^1^Control = 0, Experiment = 1; ^2^Female = 0, Male = 1; LL = Lower Level 95% Confidence Interval; UL = Upper Level 95% Confidence Interval; 10,000 bootstrapped samples used to calculate 95% confidence interval estimates. | | | | | | | |
